# Supplementary figures and images for: The role of 2,4-dihydroxyquinoline (DHQ) in Pseudomonas aeruginosa pathogenicity
Source: PeerJ. 2016 Jan 7;4:e1495. doi: 10.7717/peerj.1495 (PMC4715436; doi:10.7717/peerj.1495)

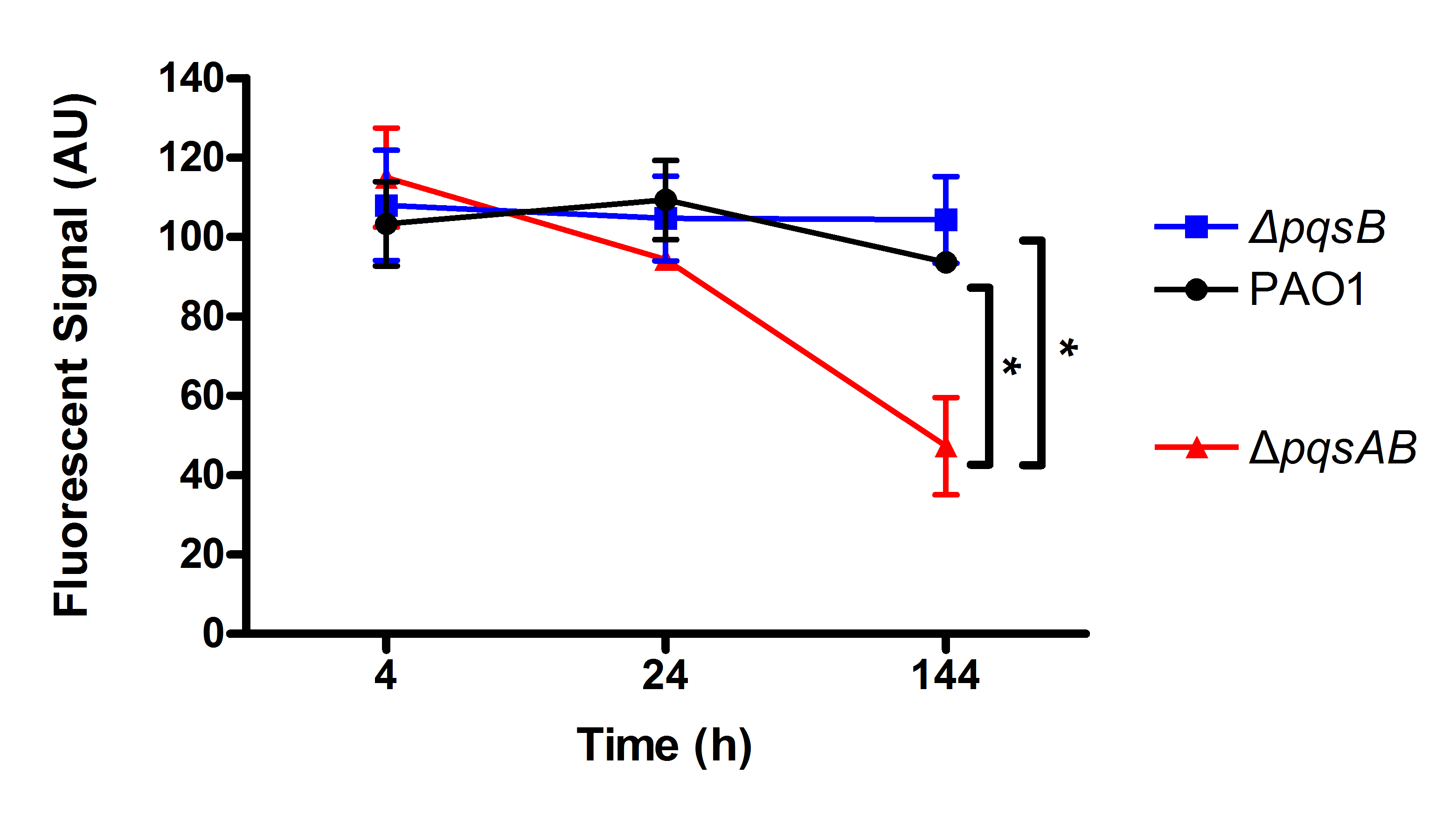

Supplement: Figure S1 — Similar amount of GFP signal was detected in the worms infected with different strains at 4 h and 24 h post infection. At 144 h time point, pqsAB mutant showed significantly reduced GFP signal (∗p-value <0.01), indicating decreased colonization of the worms by pqsAB than PAO1 and pqsB mutant. [file peerj-04-1495-s001.jpg]

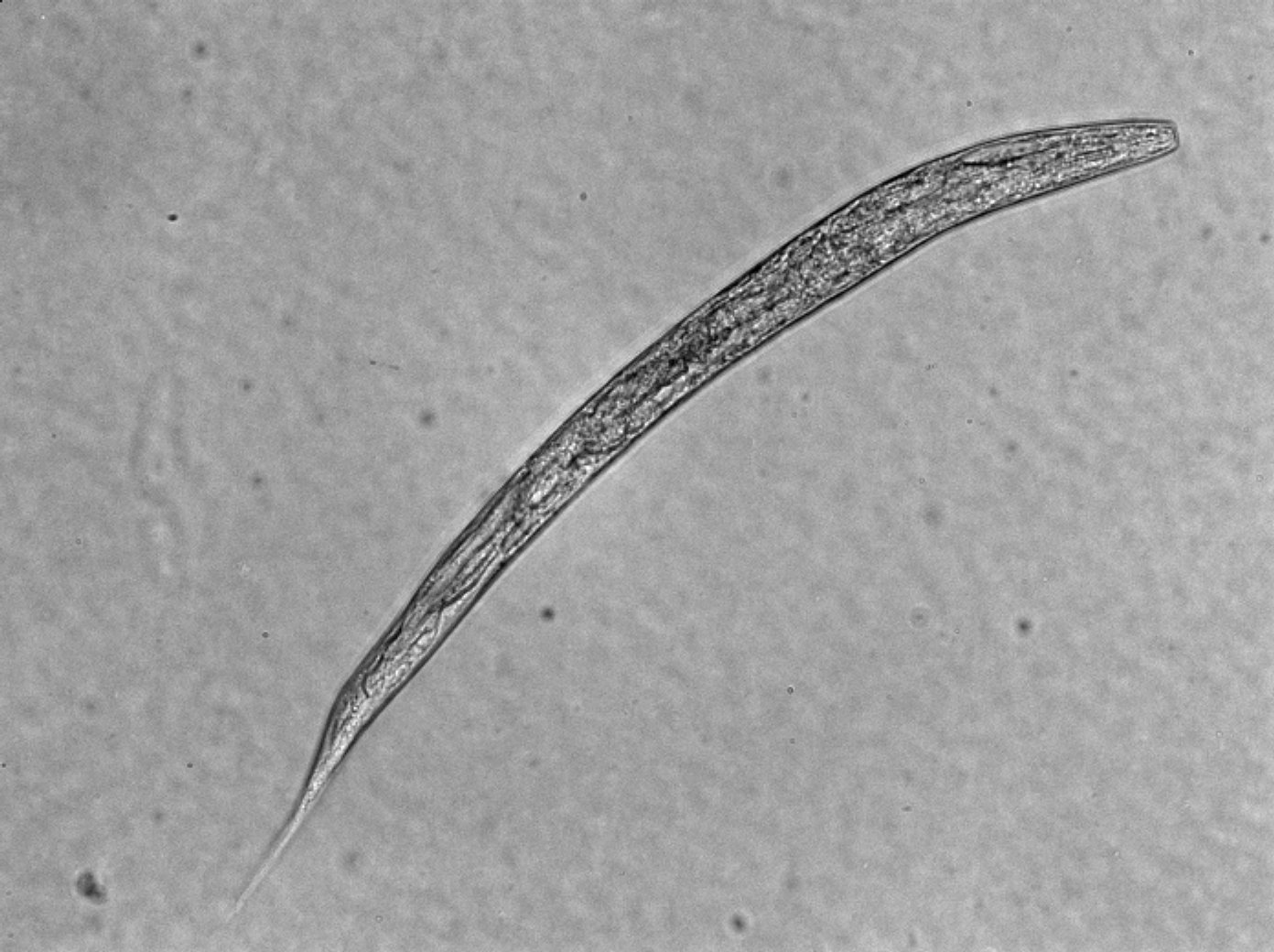

Supplement: Supplemental Information 2 — Original images from phase contrast and fluorescent microscopy of wild-type PAO1, which were used to generate Fig. 2. [file peerj-04-1495-s002.zip › PAO1 24 h BW.jpg]

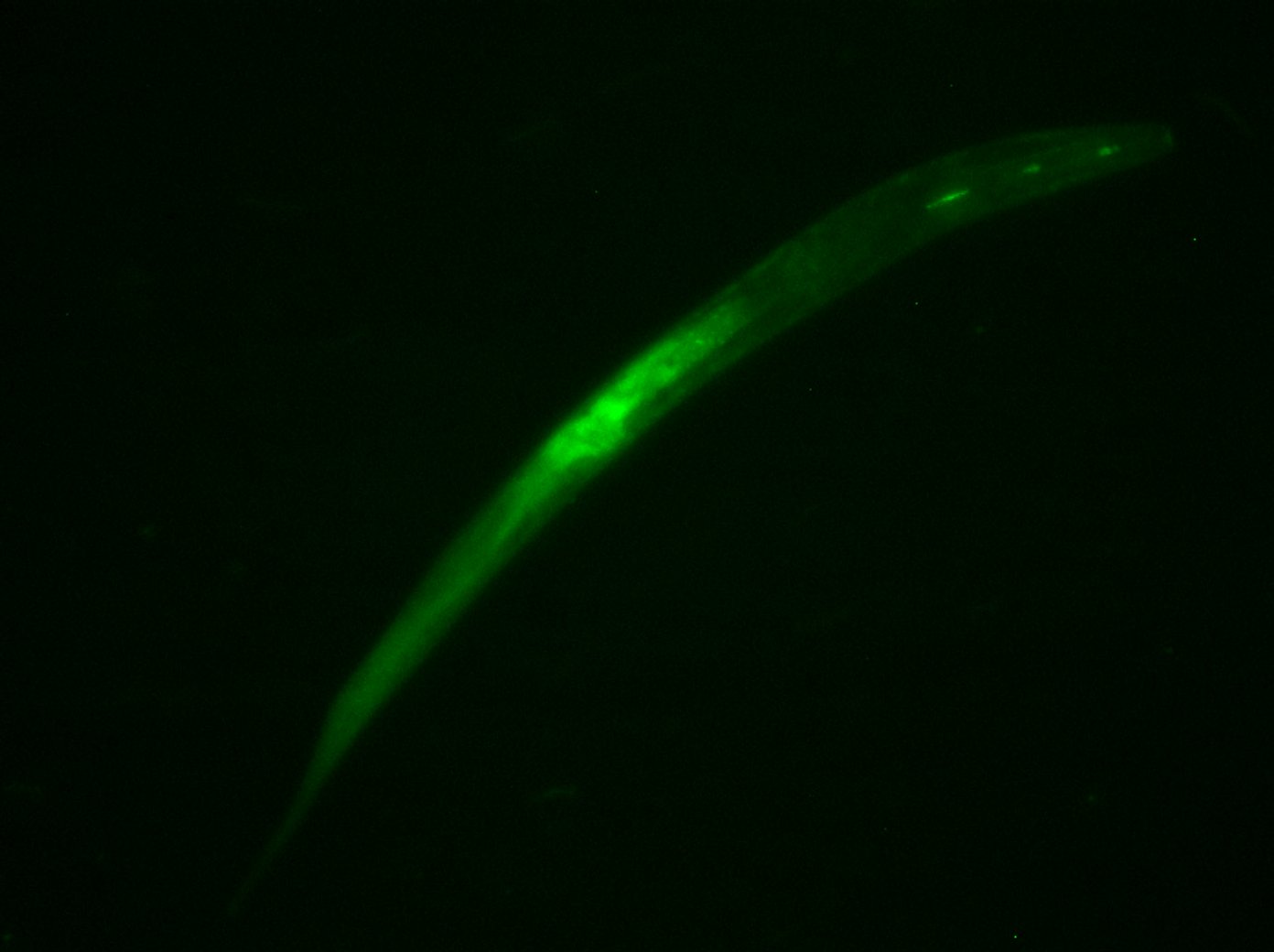

Supplement: Supplemental Information 2 — Original images from phase contrast and fluorescent microscopy of wild-type PAO1, which were used to generate Fig. 2. [file peerj-04-1495-s002.zip › PAO1 24 h GFP.jpg]

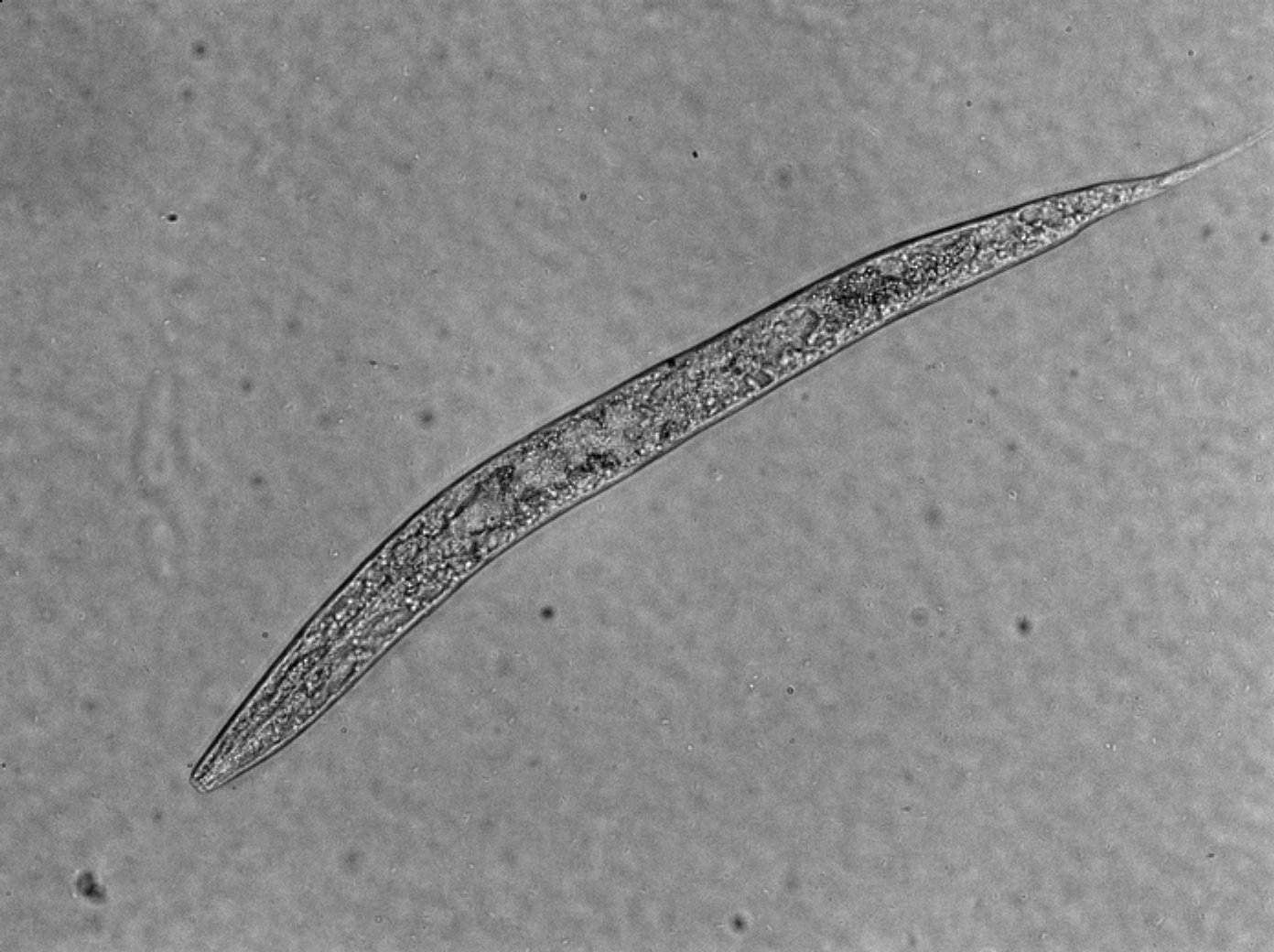

Supplement: Supplemental Information 2 — Original images from phase contrast and fluorescent microscopy of wild-type PAO1, which were used to generate Fig. 2. [file peerj-04-1495-s002.zip › PAO1 144 h BW.jpg]

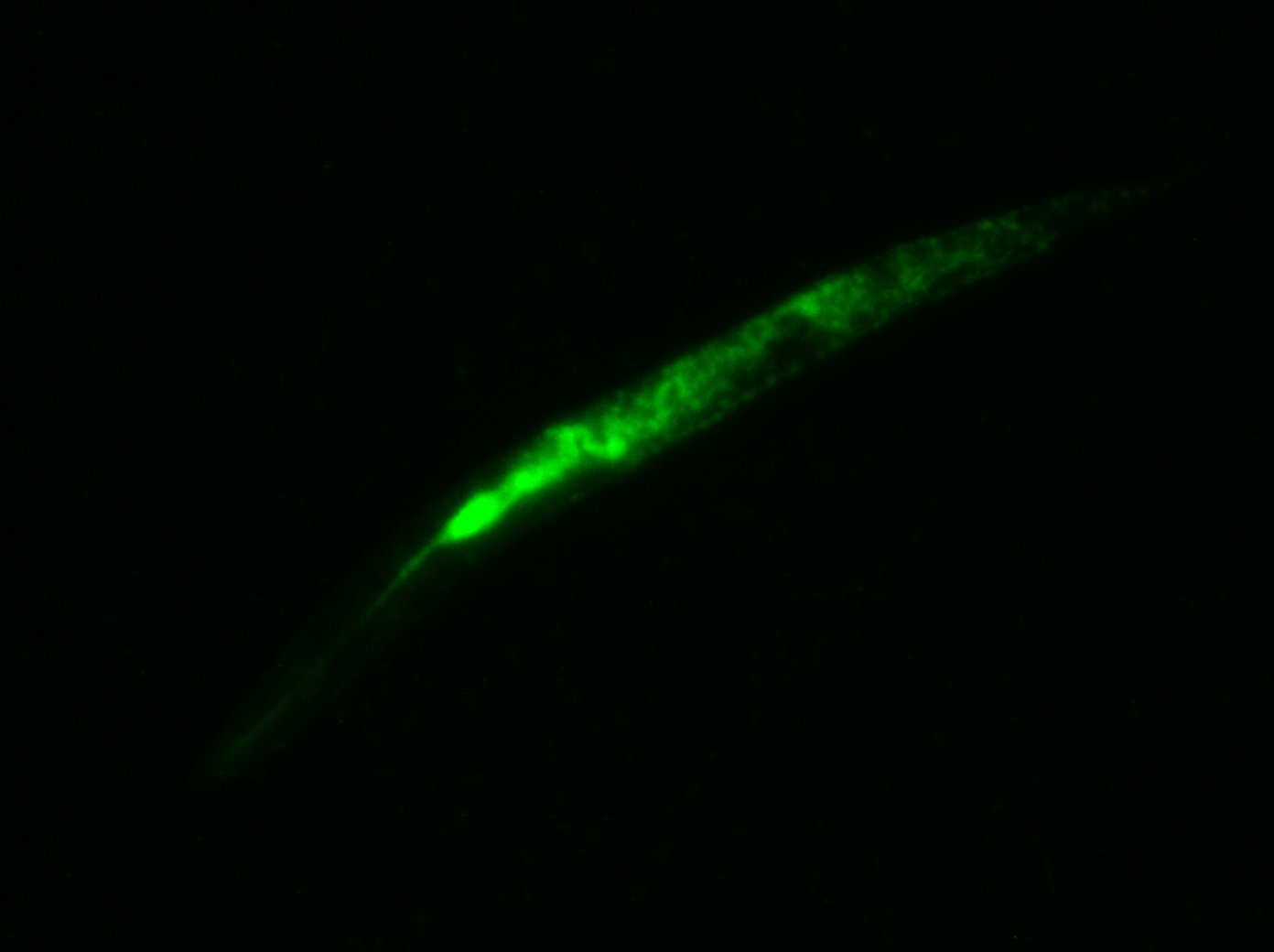

Supplement: Supplemental Information 2 — Original images from phase contrast and fluorescent microscopy of wild-type PAO1, which were used to generate Fig. 2. [file peerj-04-1495-s002.zip › PAO1 144 h GFP.jpg]

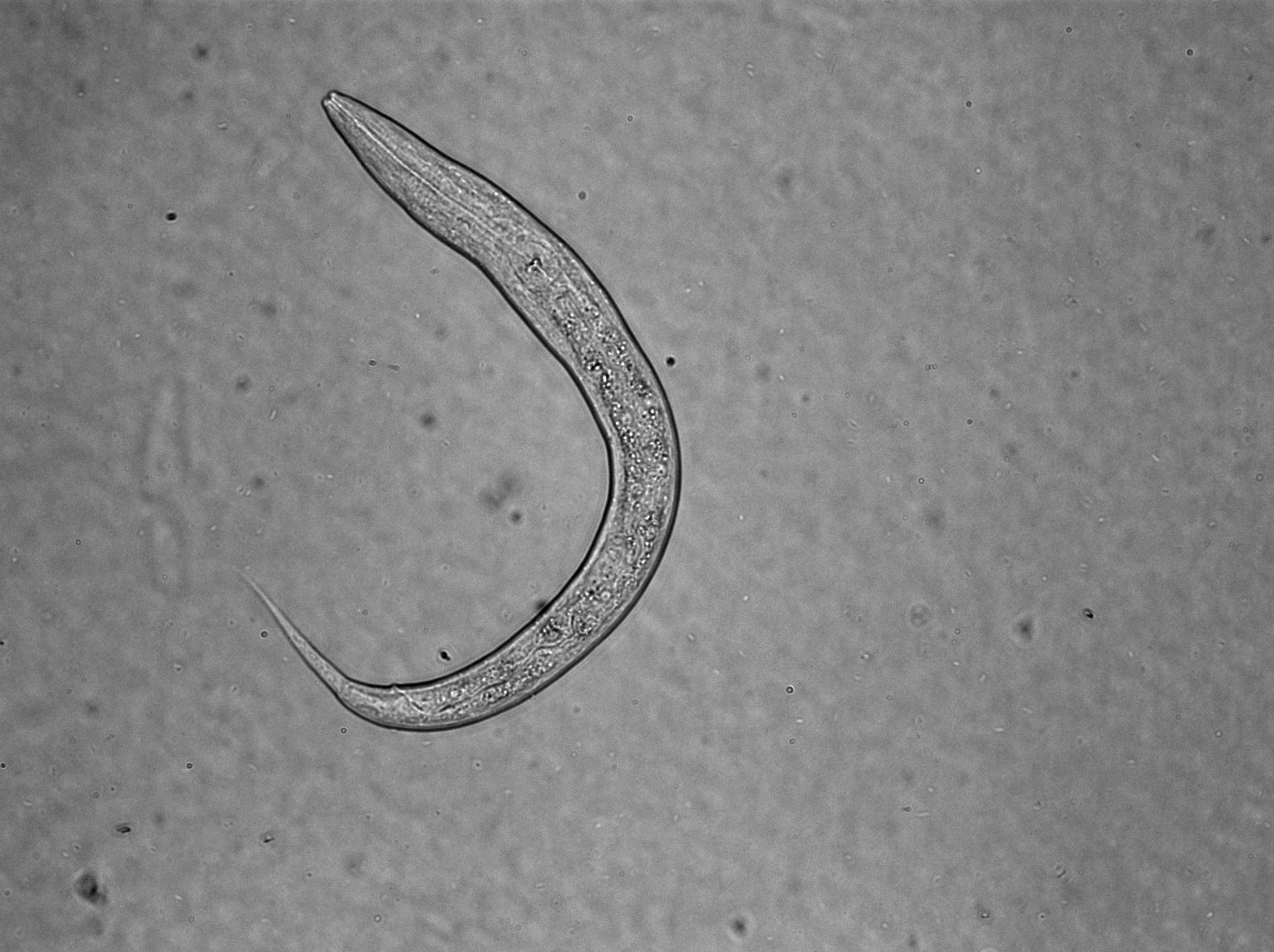

Supplement: Supplemental Information 2 — Original images from phase contrast and fluorescent microscopy of wild-type PAO1, which were used to generate Fig. 2. [file peerj-04-1495-s002.zip › PAO1 4 h BW.jpg]

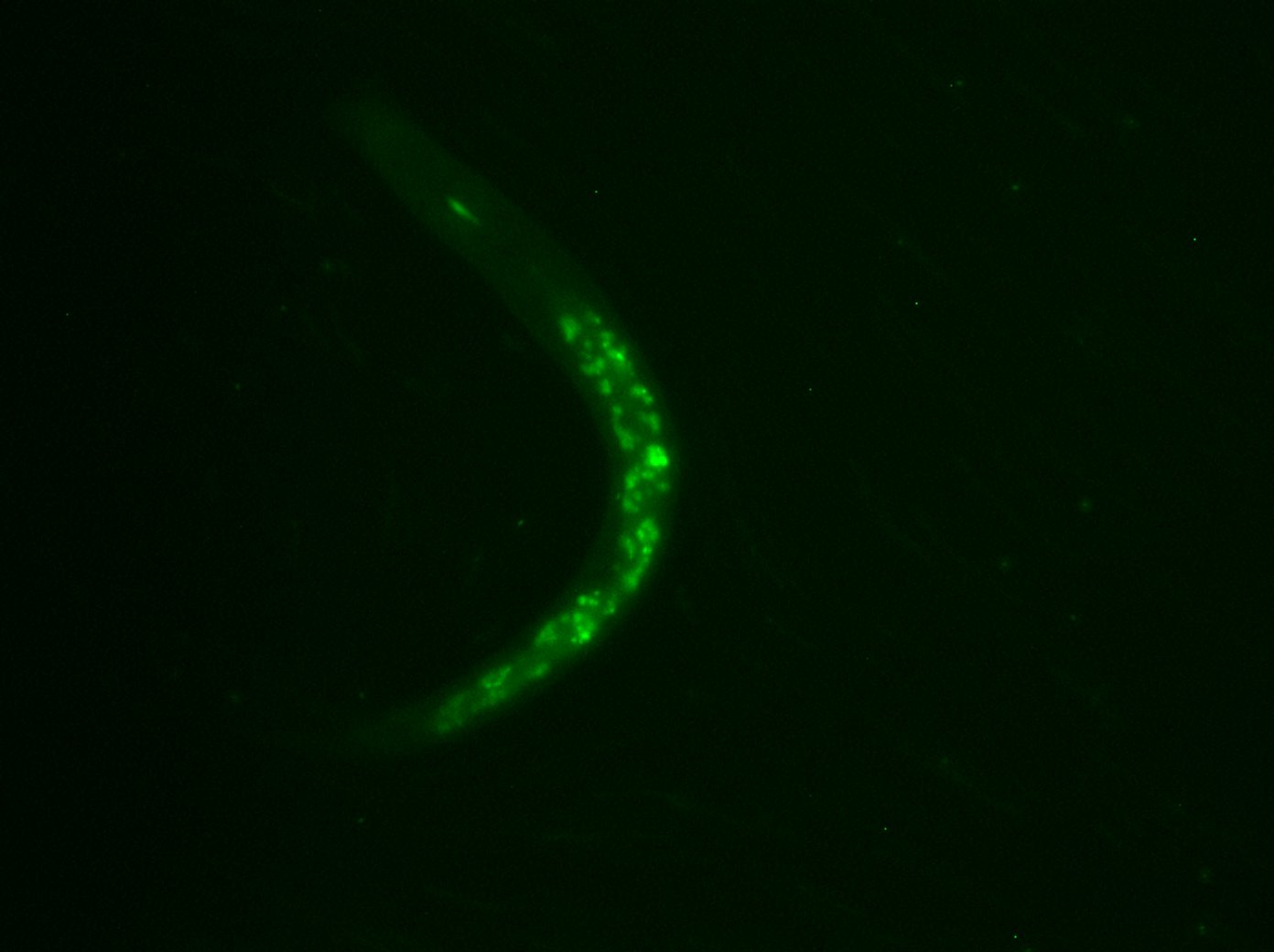

Supplement: Supplemental Information 2 — Original images from phase contrast and fluorescent microscopy of wild-type PAO1, which were used to generate Fig. 2. [file peerj-04-1495-s002.zip › PAO1 4 h GFP.jpg]

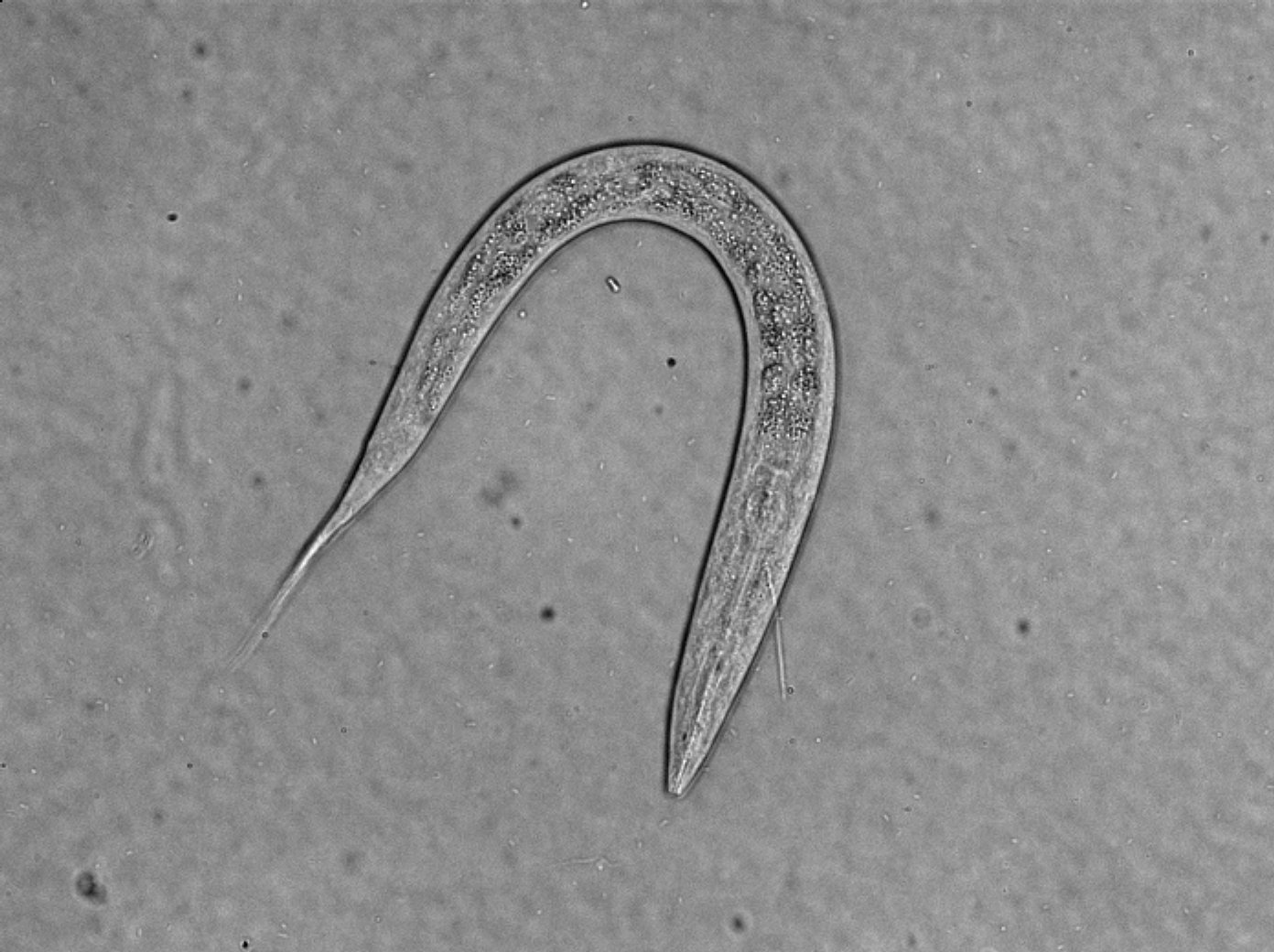

Supplement: Supplemental Information 3 — Original images from phase contrast and fluorescent microscopy of pqsB mutant, which were used to generate Fig. 2. [file peerj-04-1495-s003.zip › pqsB 24 h BW.jpg]

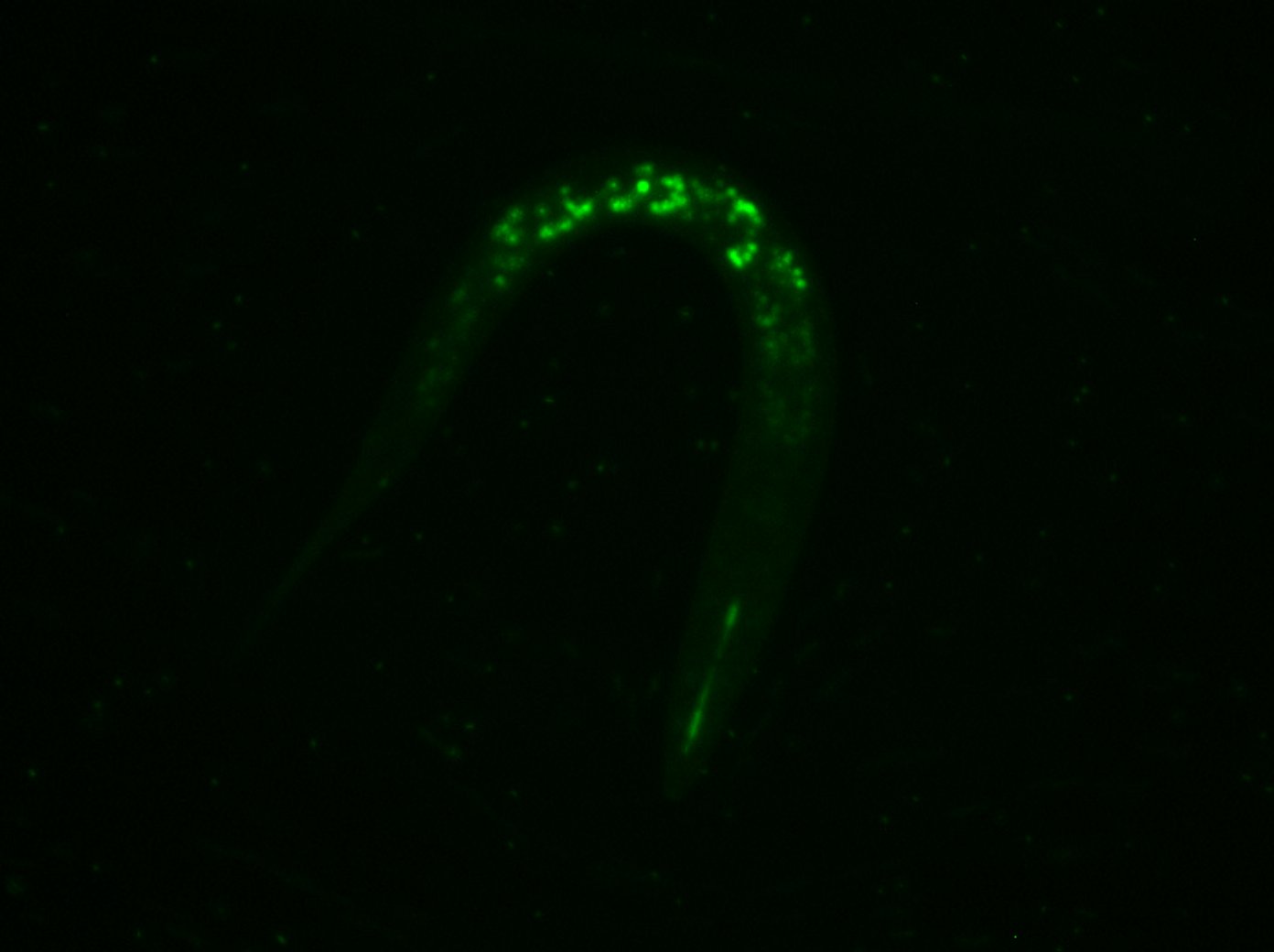

Supplement: Supplemental Information 3 — Original images from phase contrast and fluorescent microscopy of pqsB mutant, which were used to generate Fig. 2. [file peerj-04-1495-s003.zip › pqsB 24 h GFP.jpg]

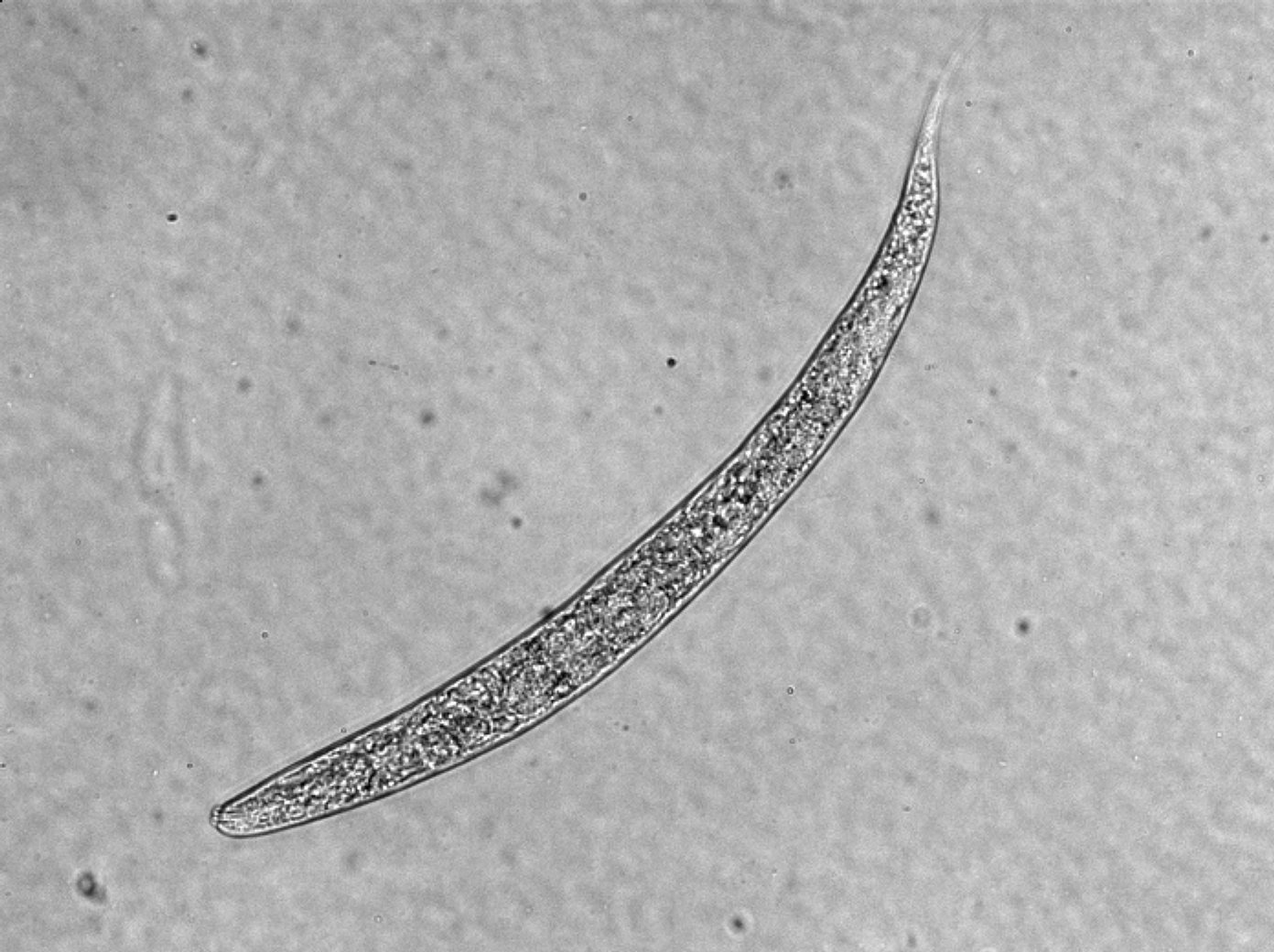

Supplement: Supplemental Information 3 — Original images from phase contrast and fluorescent microscopy of pqsB mutant, which were used to generate Fig. 2. [file peerj-04-1495-s003.zip › pqsB 144 h BW.jpg]

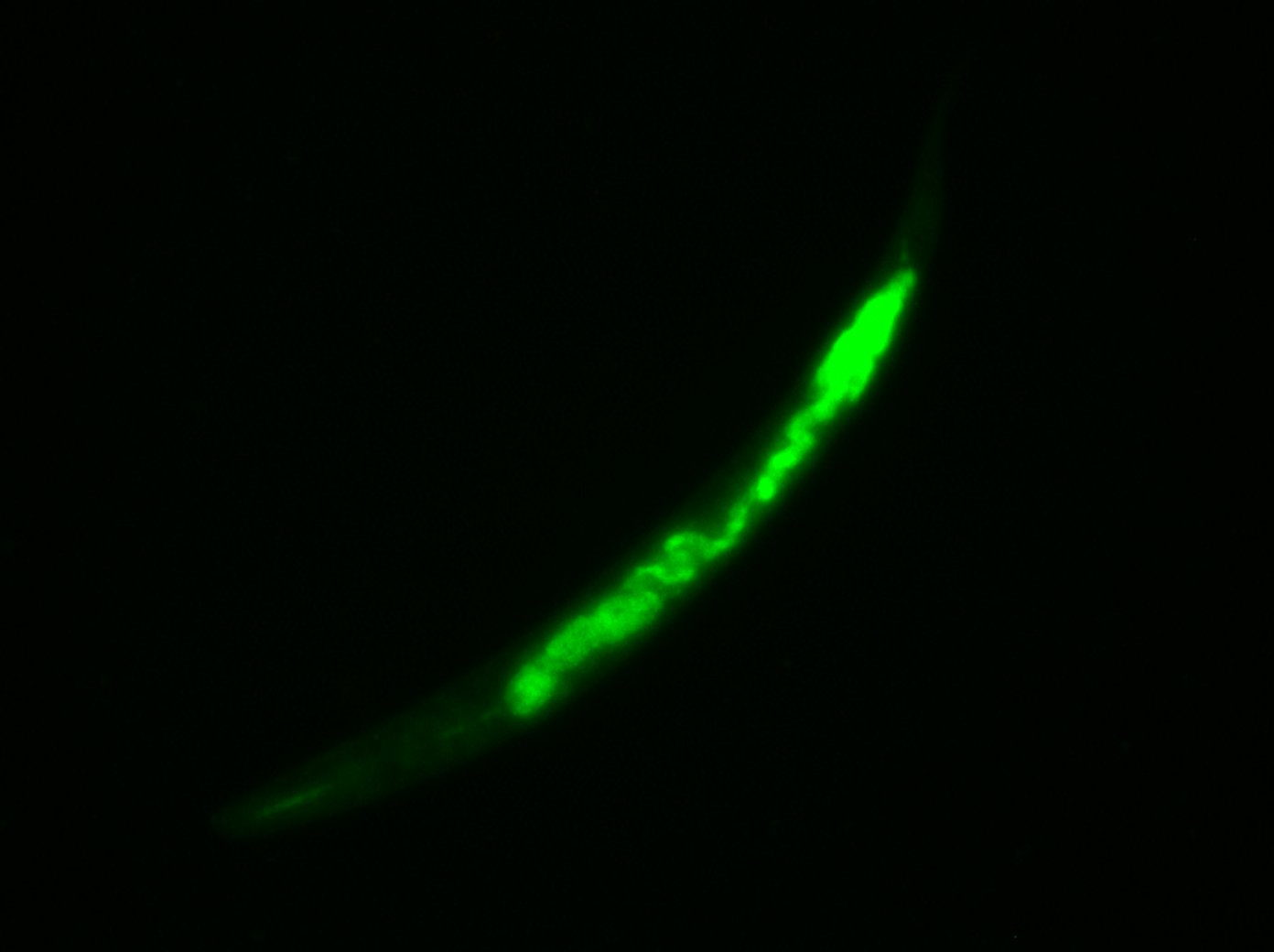

Supplement: Supplemental Information 3 — Original images from phase contrast and fluorescent microscopy of pqsB mutant, which were used to generate Fig. 2. [file peerj-04-1495-s003.zip › pqsB 144 h GFP.jpg]

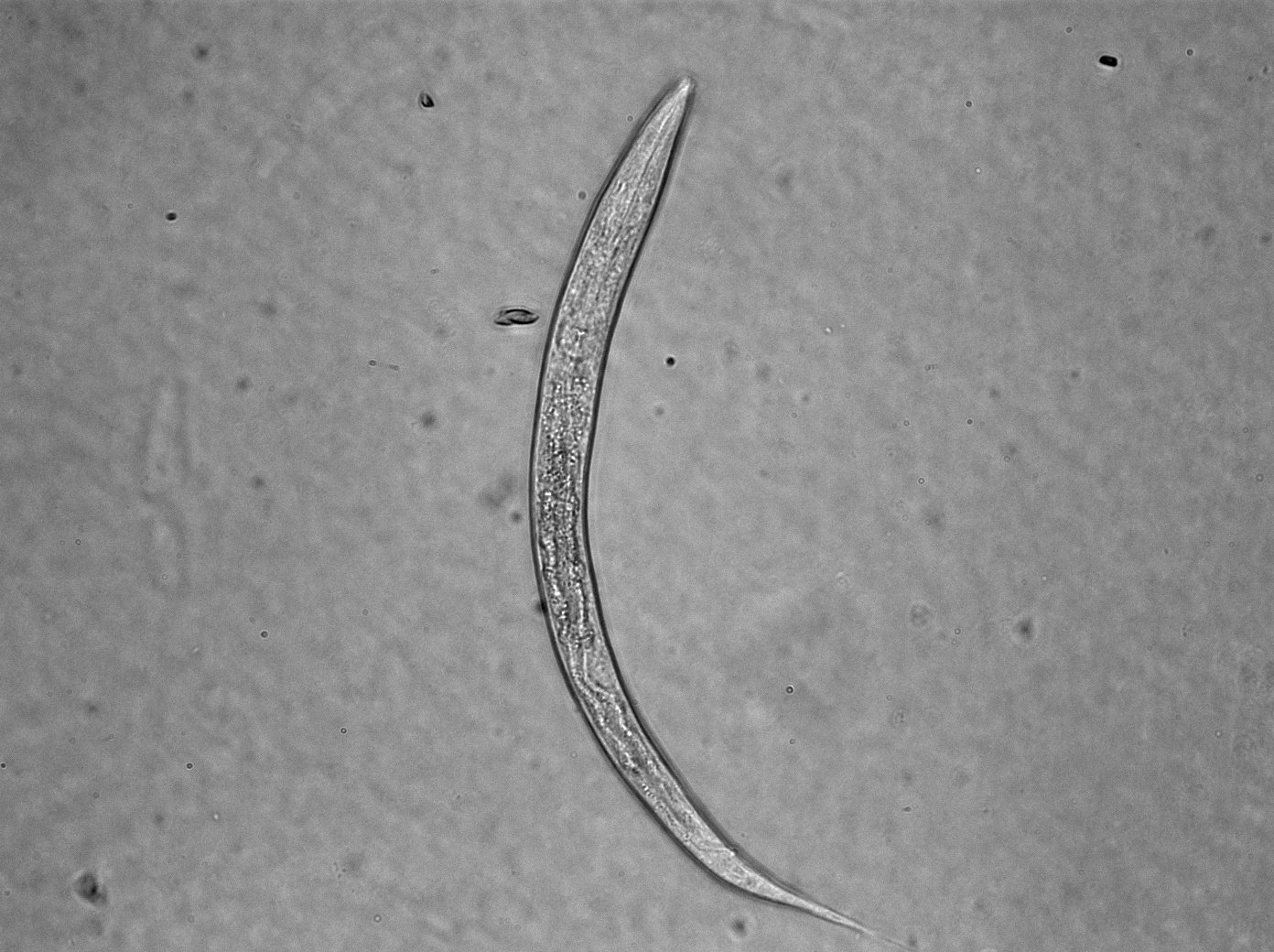

Supplement: Supplemental Information 3 — Original images from phase contrast and fluorescent microscopy of pqsB mutant, which were used to generate Fig. 2. [file peerj-04-1495-s003.zip › pqsB 4 h BW.jpg]

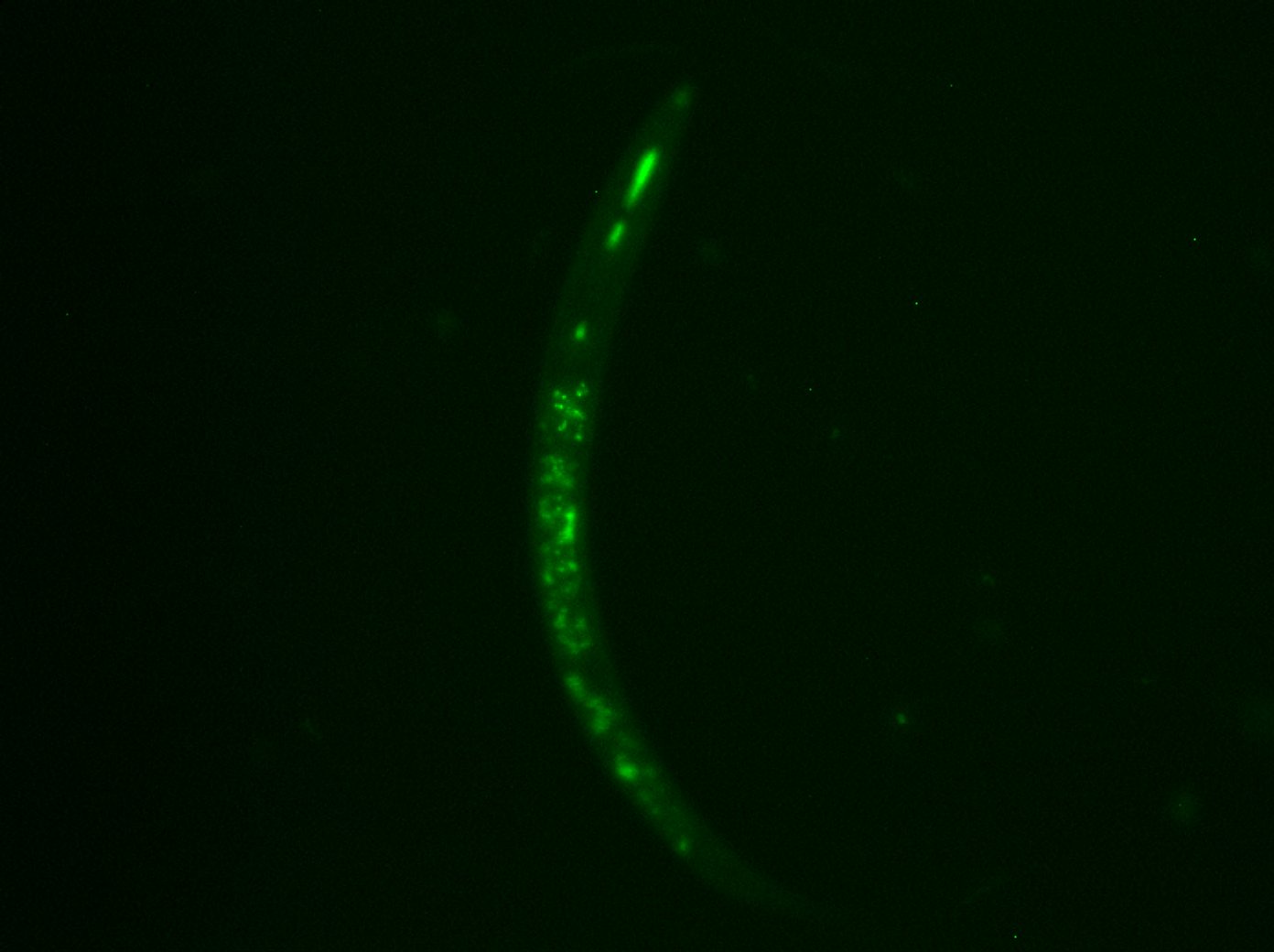

Supplement: Supplemental Information 3 — Original images from phase contrast and fluorescent microscopy of pqsB mutant, which were used to generate Fig. 2. [file peerj-04-1495-s003.zip › pqsB 4 h GFP.jpg]

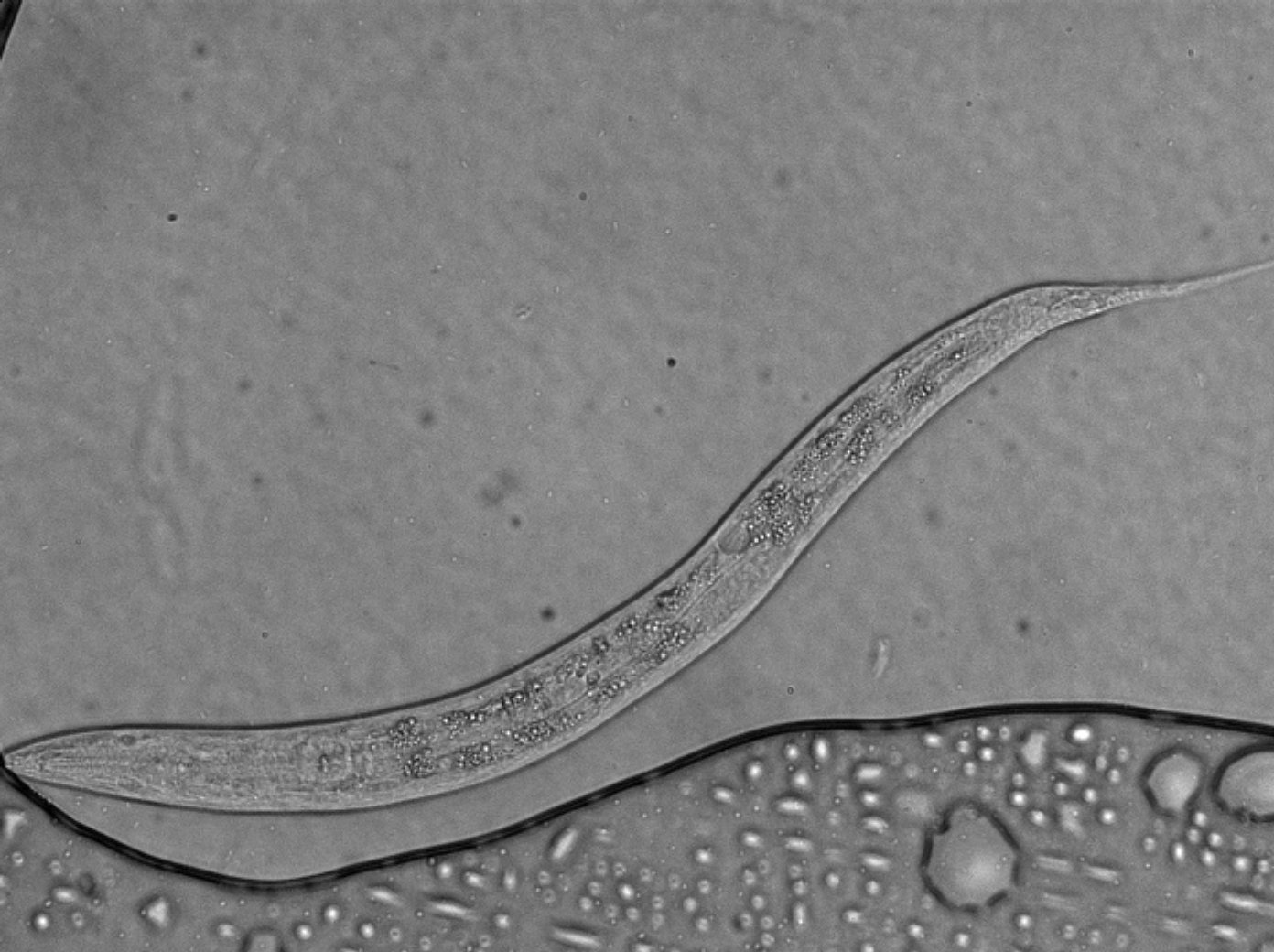

Supplement: Supplemental Information 4 — Original images from phase contrast and fluorescent microscopy of pqsAB mutant, which were used to generate Fig. 2. [file peerj-04-1495-s004.zip › pqsAB 24 h BW.jpg]

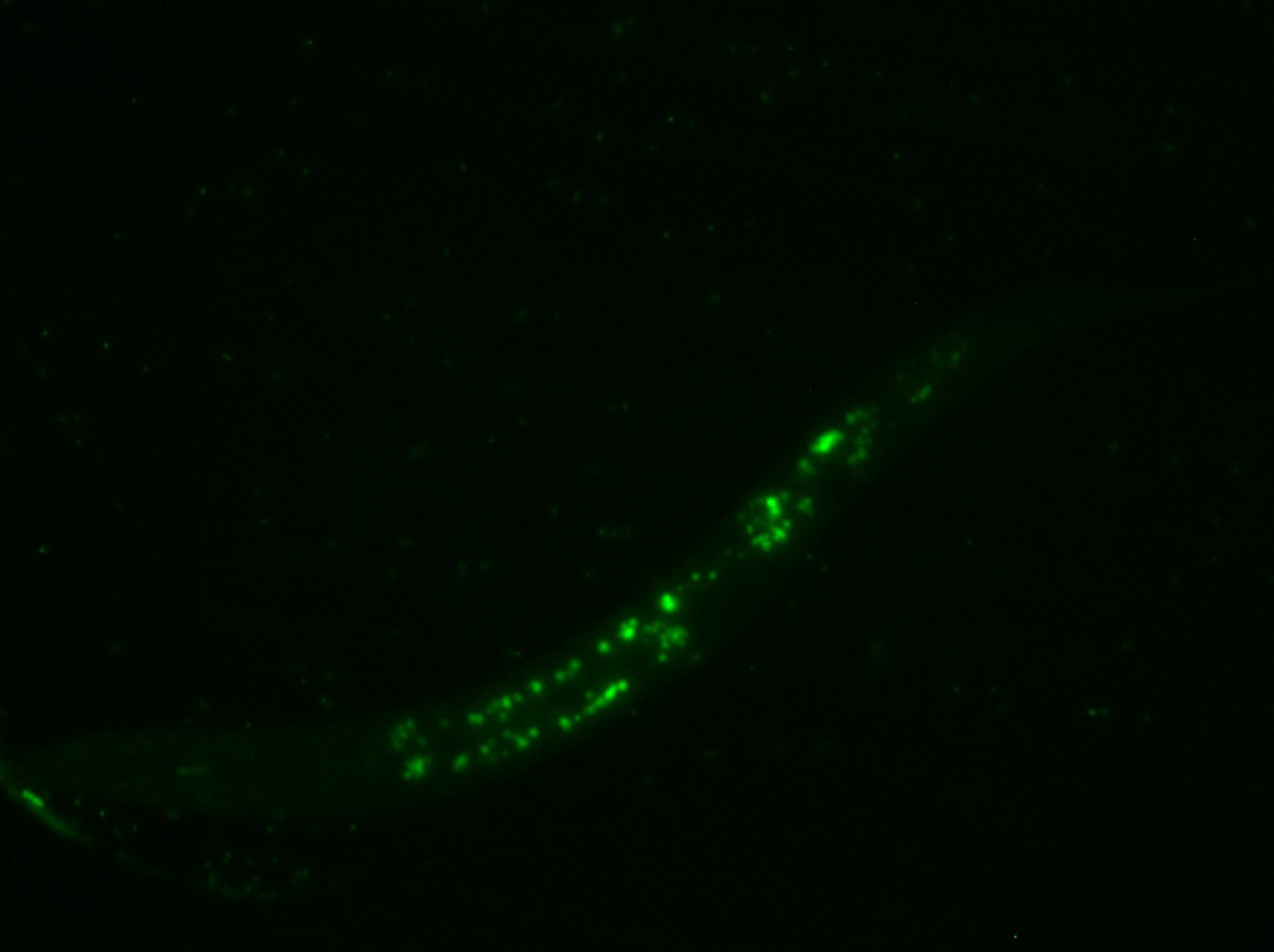

Supplement: Supplemental Information 4 — Original images from phase contrast and fluorescent microscopy of pqsAB mutant, which were used to generate Fig. 2. [file peerj-04-1495-s004.zip › pqsAB 24 h GFP.jpg]

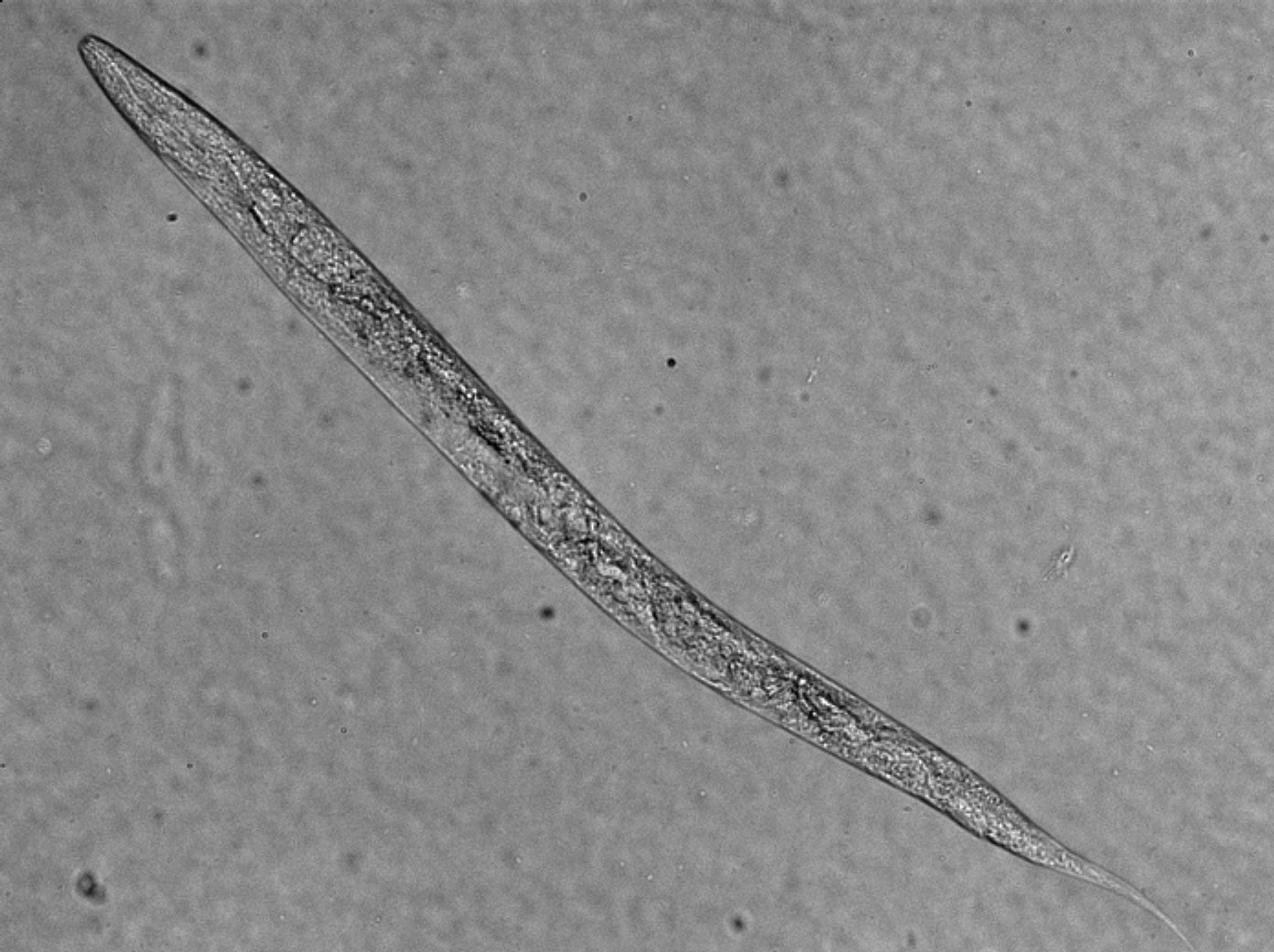

Supplement: Supplemental Information 4 — Original images from phase contrast and fluorescent microscopy of pqsAB mutant, which were used to generate Fig. 2. [file peerj-04-1495-s004.zip › pqsAB 144 h BW.jpg]

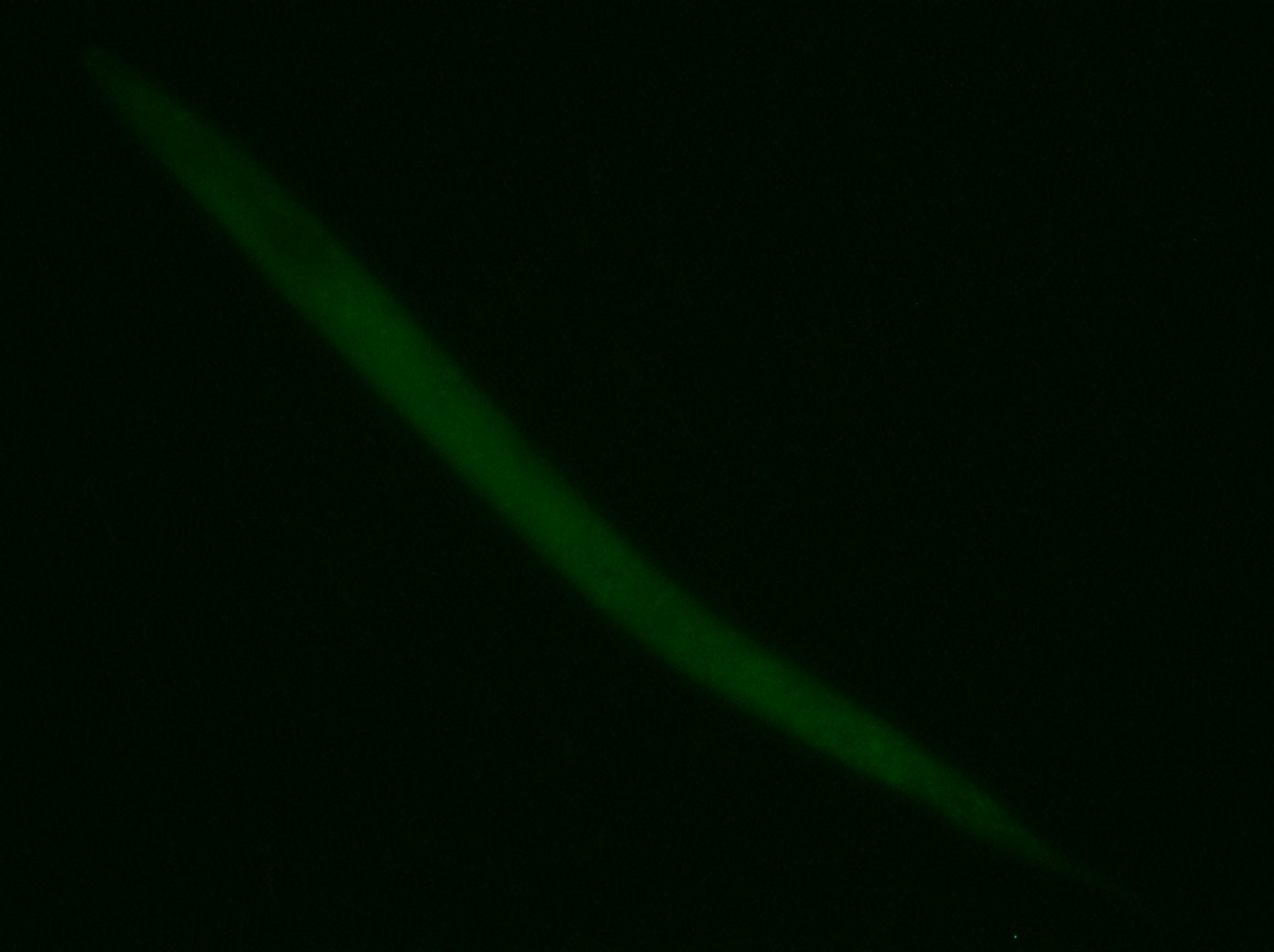

Supplement: Supplemental Information 4 — Original images from phase contrast and fluorescent microscopy of pqsAB mutant, which were used to generate Fig. 2. [file peerj-04-1495-s004.zip › pqsAB 144 h GFP.jpg]

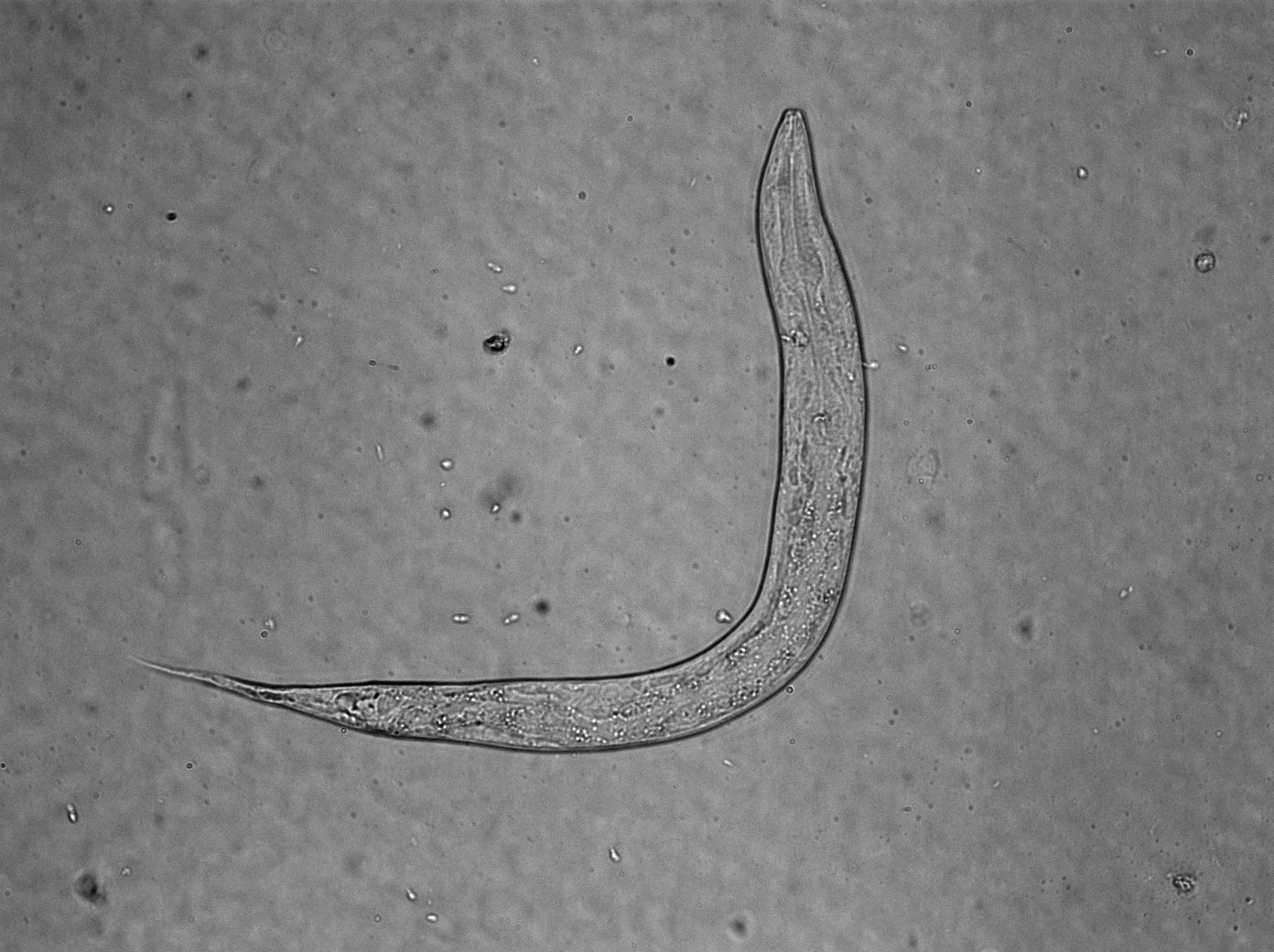

Supplement: Supplemental Information 4 — Original images from phase contrast and fluorescent microscopy of pqsAB mutant, which were used to generate Fig. 2. [file peerj-04-1495-s004.zip › pqsAB 4 h BW.jpg]

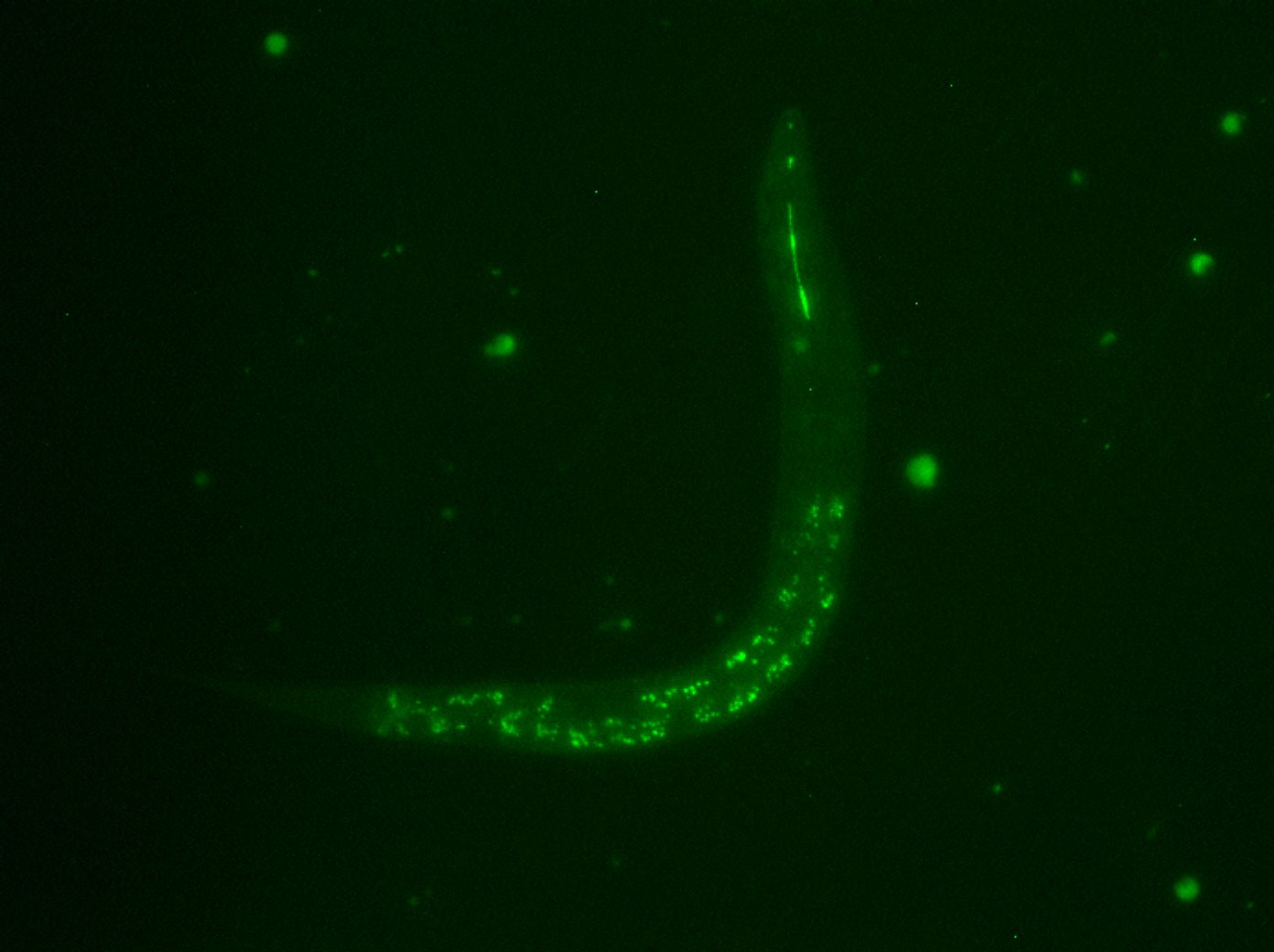

Supplement: Supplemental Information 4 — Original images from phase contrast and fluorescent microscopy of pqsAB mutant, which were used to generate Fig. 2. [file peerj-04-1495-s004.zip › pqsAB 4 h GFP.jpg]
